# Trans-grafting plum pox virus resistance from transgenic plum rootstocks to apricot scions

**DOI:** 10.3389/fpls.2023.1216217

**Published:** 2023-09-27

**Authors:** Nuria Alburquerque, Cristian Pérez-Caselles, Lydia Faize, Vincenza Ilardi, Lorenzo Burgos

**Affiliations:** ^1^ Fruit Biotechnology Group, Department of Plant Breeding, Centro de Edafología y Biología Aplicada del Segura- Consejo Superior de Investigaciones Científicas (CEBAS-CSIC), Murcia, Spain; ^2^ Research Centre for Plant Protection and Certification, Council for Agricultural Research and Economics (CREA-DC), Rome, Italy

**Keywords:** resistance, rootstock, scion, sharka, trans-grafting

## Abstract

**Introduction:**

Trans-grafting could be a strategy to transfer virus resistance from a transgenic rootstock to a wild type scion. However contradictory results have been obtained in herbaceous and woody plants. This work was intended to determine if the resistance to sharka could be transferred from transgenic plum rootstocks to wild-type apricot scions grafted onto them.

**Methods:**

To this end, we conducted grafting experiments of wild- type apricots onto plum plants transformed with a construction codifying a hairpin RNA designed to silence the PPV virus and studied if the resistance was transmitted from the rootstock to the scion.

**Results:**

Our data support that the RNA-silencing-based PPV resistance can be transmitted from PPV-resistant plum rootstocks to non-transgenic apricot scions and that its efficiency is augmented after successive growth cycles. PPV resistance conferred by the rootstocks was robust, already occurring within the same growing cycle and maintained in successive evaluation cycles. The RNA silencing mechanism reduces the relative accumulation of the virus progressively eliminating the virus from the wild type scions grafted on the transgenic resistant PPV plants. There was a preferential accumulation of the 24nt siRNAs in the scions grafted onto resistant rootstocks that was not found in the scions grafted on the susceptible rootstock. This matched with a significantly lower relative accumulation of hpRNA in the resistant rootstocks compared with the susceptible or the tolerant ones.

**Discussion:**

Using transgenic rootstocks should mitigate public concerns about transgenes dispersion and eating transgenic food and allow conferring virus resistance to recalcitrant to transformation cultivars or species.

## 1 Introduction

Sharka is the most severe viral disease in trees of the *Prunus* genus, causing substantial economic losses (Rodamilans et al., 2020). The etiological agent is the Potyvirus *Plum pox virus* (PPV) which is naturally transmitted by aphids in a non-persistent manner and by grafting PPV-infected material.

PPV has great genetic diversity. PPV isolates can be subdivided into ten strains based on phylogenetic analyses (Hajizadeh et al., 2019; Rodamilans et al., 2020). Among these strains, the two most widespread and economically significant are Dideron, PPV-D and Marcus, PPV-M (García et al., 2014). In particular, PPV-D is endemic in Spain. Symptoms induced by PPV on apricot include chlorotic bands and rings on leaves and stones, fruit deformation, and early fruit drops devaluating fruits (Agustí, 2010). The virus does not kill the trees but may largely reduce their production (García and Cambra, 2007).

PPV resistance presents one of the most discussed topics in European apricot (*Prunus armeniaca* L.) breeding programs (Rubio et al., 2008; Krška, 2018). All apricot cultivars of European origin are susceptible to PPV (Krška, 2018). One major limitation of introducing PPV resistance in European apricots is that most of the genetic sources are North American cultivars (Martínez-Gómez et al., 2000), characterized by high chilling requirements and poorly adapted to the Mediterranean climatic conditions. In this context, exploring alternative strategies to confer PPV resistance is essential.

Among the transgenic strategies used to induce PPV resistance, significant results have been obtained through the biotechnological exploitation of RNA silencing (reviewed in Ilardi and Tavazza, 2015). RNA silencing is a sequence-specific gene-regulation mechanism widely conserved among eukaryotes. In plants, among other functions, RNA silencing exerts a pivotal defense role against viruses and viroids. Double-stranded RNA (dsRNA) is the crucial trigger for RNA silencing. Dicer, a ribonuclease (RNase) III family, is involved in the cleavage of the dsRNA into 21–24 nucleotide (nt) duplexes, referred to as small interfering RNAs (siRNAs). In the case of Post-Transcriptional Gene Silencing (PTGS), siRNAs loaded into Argonaute proteins guide them to slice and/or translational repress complementary RNA sequences (reviewed in Zhao and Guo, 2022). Transgenic expression of a viral-derived dsRNA has been proven to be a robust strategy to confer virus resistance in crops (Smith et al., 2000). RNA silencing can spread to neighboring cells through plasmodesmata or systemically through the vascular system (Palauqui et al., 1997; Mlotshwa et al., 2002). Thus, RNA silencing induced in a restricted tissue of the plant can potentially spread to other tissues. The nature of the mobile RNA silencing signals remained debated until two complementary works shed light on it (Dunoyer et al., 2010; Molnar et al., 2010). Using different approaches, they showed that siRNAs are indeed the mobile signal. The evidence that RNA silencing can move over long distances through the vascular system opened the question of whether virus resistance of a transgenic rootstock could be transmitted to a non-transgenic scion through grafting, thus overcoming the concern about the spreading of transgenes in the environment and eating transgenic products (Arpaia et al., 2020).

Grafting has been traditionally used to join scions and rootstocks of fruit trees with different genomes. In apricot, as in most fruit trees, it is a common practice for vegetative propagation of commercial cultivars. Additionally, it is commonly employed for horticultural crops such as tomatoes or cucurbits to improve productivity (Melnyk and Meyerowitz, 2015).

The early evidence on the applicability of transgenic rootstock:wt scion (TR : WS) grafting to confer viroid resistance came from the work of Kasai et al. (2013). They showed that genetically modified tobacco rootstocks expressing *Potato spindle tuber viroid* (PSTVd) siRNAs could attenuate PSTVd accumulation in a non-genetically modified tobacco scion grafted on the stock. In subsequent work, wild-type tomatoes partially resistant to *Cucumber mosaic virus* (CMV) were obtained after grafting them onto transgenic tomatoes expressing intron-spliced hairpin RNA (ihpRNA) designed to silence different CMV genes (Bai et al., 2016). Similarly, Zhao and Song (2014) showed that *Prunus necrotic ring spot virus* (PNRSV) hpRNA-derived siRNAs from the transgenic cherry rootstocks could confer a certain degree of resistance to the non-transgenic sweet cherry scions. However, despite this encouraging evidence, contradictory results were also obtained in herbaceous and woody plants (Leibman et al., 2015; Sidorova et al., 2021). In particular, Sidorova et al. (2021) showed that although the transgenic rootstocks of the interspecific Elita cv. [(*Prunus pumila* L. × *P. salicina* Lindl.) × (*P. cerasifera* Ehrh.)] accumulate a high level of PPV coat protein (CP) specific siRNAs, the trans-grafting was not successful in promoting PPV resistance in non-transgenic scions.

However, besides transgenic constructs using PPV CP sequences, significant results were obtained with the h-UTR/P1 construct, which encodes an ihpRNA encompassing the first 733 nt of the PPV-M ISPaVe44 genome (Di Nicola-Negri et al., 2005). In model plants, h-UTR/P1 induced long-lasting PPV resistance not only to the homologous ISPaVe44 isolate but also to isolates belonging to D, M, Rec, and the distantly related EA and C PPV strains (Di Nicola-Negri et al., 2005; Di Nicola-Negri et al., 2010). In addition, authors showed that PPV resistance was maintained in transgenic *Nicotiana benthamiana* plants under biotic and abiotic stresses (Di Nicola et al., 2014). Notably, when the h-UTR/P1 construct was introduced in the plum, 70% of clones were resistant to PPV in *in vitro* and greenhouse (García-Almodóvar et al., 2015). These encouraging data prompt us to determine if the resistance to sharka could be transferred from the transgenic plum rootstocks to wild-type apricot scions grafted onto them. To this end, we conducted grafting experiments of wild- type apricots onto the transgenic plum rootstocks and studied if the resistance was transmitted from the rootstock to the scion.

## 2 Materials and methods

### 2.1 Transgenic plant material used in the study

Four transgenic plum (*Prunus domestica* L.) lines St5’-1, St5’-6, St5’-7, and St5’-9, obtained after transforming ‘Stanley’ hypocotyls and with different resistance levels to PPV (García-Almodóvar et al., 2015), were used in the study. Lines St5’-1 and St5’-9 are highly PPV resistant since the virus was never found in any plant. Line St5’-6 is considered tolerant because the virus was detected early after graft-mediated PPV inoculation but was undetectable later. Transgenic line St5’-7 is PPV-susceptible since most of the challenged plants were infected with the virus (García-Almodóvar et al., 2015).

*In vitro* transgenic plum shoots were multiplicated, rooted, and acclimatized in the greenhouse and successively transplanted to 5L forestall pots to allow for a good root system development. After one year (two growing cycles) plants were large enough to try grafting them.

### 2.2 Graft-mediated PPV inoculation

To evaluate if RNA silencing-mediated resistance to PPV found in St5’-1, St5’-6, and St5’-9 transgenic plums can be transmitted to apricots grafted onto them, we utilized as inoculum source the Spanish PPV isolate 3.3 RB/GF-IVIA (AF172346.1) belonging to the PPV-D strain (García-Almodóvar et al., 2015), adopting two different virus inoculation procedures.

**Figure 1:**
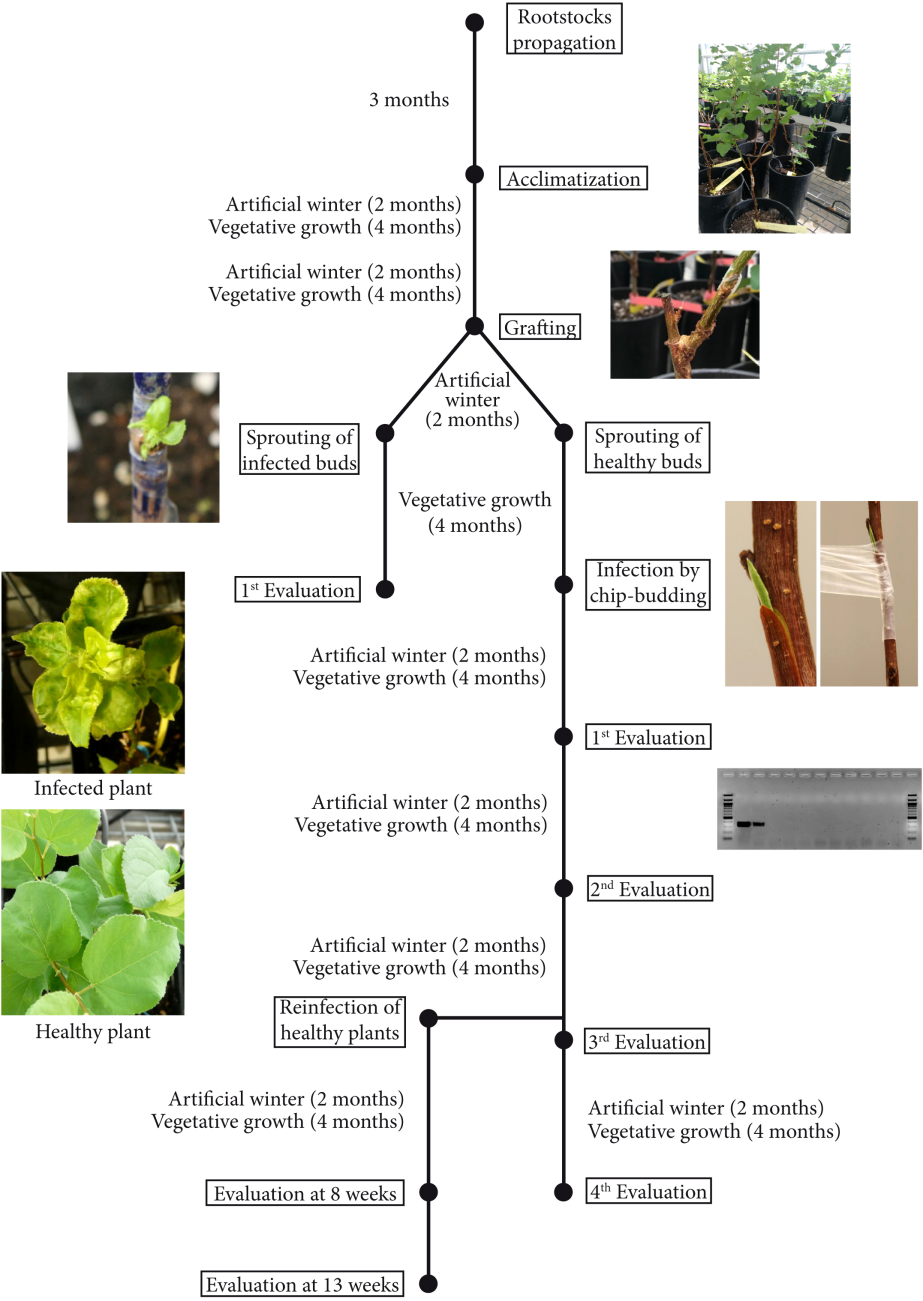
Schematic drawing of the four years of sharka resistance evaluation by RT-PCR in wild type apricots grafted onto transgenic plum rootstocks. All evaluations were performed at 8 weeks of vegetative growth after an artificial winter, at least indicated otherwise.

First, wild-type (wt) ‘Canino’ apricot buds infected by PPV and with clear sharka symptoms were grafted onto the transgenic plum rootstocks (**Supplementary Figure 1**), exposed to a two-month artificial winter in the cold chamber, and then transferred to the greenhouse. Eight weeks later, sprouted buds were evaluated for virus resistance (see below). In the second experimental setting, healthy ‘Canino’ apricot buds (two-three) were grafted onto the transgenic plum rootstocks. After an artificial winter and sprouting of the buds, the scions were inoculated by chip-budding with new shoots of PPV-infected GF305 peaches (**Supplementary Figure 2**). At the end of the cycle, plants were again transferred to the cold chamber and evaluated for virus resistance in the following cycle, as described above (**Figure 1**).

### 2.3 Evaluation of virus infection

Healthy grafted plants were infected by chip-budding (**Supplementary Figure 2**) and subjected to successive growth cycles in the greenhouse and artificial winter in a cold chamber. After two months at 7 °C in the cold chamber, plants were transferred to the greenhouse. After eight weeks, plants were evaluated by RT-PCR. Young leaves (100 mg from the third or fourth leaf) from each plant were collected, quickly frozen in liquid nitrogen, and maintained at -80°C until use. Plant RNA extraction was performed using the commercial “NucleoSpin® RNA Plant and Fungi” (Machery-Nagel, Düren, Germany), following manufacturer instructions. 1.5 µg of RNA (the volume depends on RNA concentration) was mixed with 1 µl 10 mM oligo-dT primers (final volume 15 µl), and heated at 70°C for 5 minutes, then 10 µl of the buffer M-MLV 5x containing retro transcriptase and dNTPs was added, and the mixture was heated for one hour at 42°C. First-strand cDNA was used as the template in PCR reactions to amplify a 313 bases pair-fragment of the PPV capsid gene using primers VP337 (CAATAAAGCCATTGTTGGATC) and VP338 (CTCTGTGTCCTCTTCTTGTG) (Martínez-Gómez et al., 2003) in a final volume of 25 µl containing 12.5 µl of GoTaq® Green Master Mix buffer (Promega). Thermocycling was performed using a 2 min heating step at 94°C followed by 35 cycles of 94°C for 30 s, 58°C for 30 s, and 72°C for 30 s, followed by a final extension of 10 min at 72°C.

### 2.4 Northern blot for small interference RNAs

For siRNA analysis, total RNA was extracted from transgenic and untransformed leaves with the Tri® Reagent (Sigma-Aldrich Co., St. Louis, Mo, USA), following manufacturers’ directions. Samples equivalent to 20 µg of total RNA, as evaluated by Nanodrop® analysis (NJ1000 Nanodrop Technology Inc., Wilmington, DE, USA), were dissolved in 50% formamide and, after heat-treatment, were loaded onto a denaturing polyacrylamide gel (17,5%). After electrophoresis, the nucleic acids were electro-blotted to a positively charged nylon membrane (Roche Applied Science, Mannheim, Germany). Hybridization was performed in Dig Easy Hyb buffer (Roche Applied Science, Mannheim, Germany) using a 780-bp DIG-labeled RNA probe corresponding to part of the PPV sequence present in the h-UTR/P1 construct (Di Nicola-Negri et al., 2005). This DNA fragment was PCR-amplified with specific primers (**Supplementary Figure 3**) 35Sdag5’-F (TTCGCAAGACCCTTCCTCTA) and 35Sdag5’-R (CCATTTGCCTTAGCGTTTGT) and cloned within the pGEM^®^-T Easy Vector (Promega,Madison,WI,USA) and the orientation was checked to select restriction enzymes and RNA polymerase for transcription. DIG RNA probe labeling was performed following manufacturer kit instructions (Roche, Mannheim, Germany). For siRNAs detection, membranes were treated with anti-DIG antibody-alkaline phosphatase and CDP-Star (Roche Applied Science, Mannheim, Germany) and exposed to chemiluminescence (AmershamTM Imager 600).

### 2.5 Estimation of the relative amount of the virus and h-UTR/P1 hpRNA

To estimate the relative amount of the virus in apricot scions grafted onto the four transgenic plum rootstocks, RNA extraction was performed from plants, which were revealed to be RT-PCR positive.

Leaves harvested were snap-frozen in liquid nitrogen and stored at − 80°C until use. Plant RNA extraction was performed using the commercial “RNeasy Plant Mini Kit” (Qiagen), following manufacturer instructions. RNAs were digested with DNase I using the DNA-free Kit (Ambion, Austin, TX, USA) and quantified using a spectrophotometer Nanodrop ND-1000 (Nanodrop Technologies, Wilmington, USA). cDNA was synthesized using the RETROscript cDNA Synthesis Kit (Promega, Madison, WT, USA) following manufacturer instructions. PPV concentration was established by real-time RT-PCR using the GeneAmp 7500 sequence detection system (Applied Biosystems, Foster City, CA, USA). The EF-1 α gene was used for the normalization. The cDNA was synthesized as described above, and the qPCR was carried out using the SYBR Green Master Kit (Applied Biosystems).

Primers used in this study were PPV-U (TGAAGGCAGCAGCATTGAGA) and PPV-RR (CTCTTCTTGTGTTCCGACGTTTC) to amplify PPV, designed by Varga and James (2005), and q35S5’-F (CTCATTCACTTGCCACCTCG) and q35S5’-R (ATGCACGTTACTGACTTGGC) for the transgene h-UTR/P1 hpRNA, specifically designed for this study (**Supplementary Figure 3**). For the housekeeping EF-1 α gene amplification primers TEF2-f (GGTGTGACGATGAAGAGTGATG) and TEF2-r (TGAAGGAGAGGGAAGGTGAAAG) were used. Each set of primers was mixed at a final concentration of 300 nM with 2 µl cDNA and 1 × SYBR Green. After denaturation at 95°C for 10 min, a two-step procedure of 15 s denaturation and 1 min of annealing and extension at 60°C for 40 cycles was adopted. These conditions were used for target and reference genes, and the absence of primer dimers was checked in controls lacking templates. Each biological replicate was a pool of three different plants, and three biological replicates were used. For each biological replicate, six technical replicates were run. For the calculation of relative virus content, the 2^-ΔΔCt^ method was used (Livak and Schmittgen, 2001). The Ct value was adjusted automatically, and the threshold cycle value difference (ΔCt) between the Ct of the target gene (virus or transgene) and Ct of EF-1 α (internal control) was used to normalize the amount of the genes. As long as the target genes and the internal control have similar amplification efficiencies, Ct values were normalized using the difference (ΔCt) between the internal control and target genes. This value is calculated for each sample to be quantified. Finally, the relative quantification of the virus gene and transgene hpRNA in each sample was calculated according to the formula where the reference sample was the susceptible line St5’-7. Relative quantification = 2^−ΔΔCt^, where ΔΔCt = ΔCt (unknown sample) - ΔCt (reference sample).

### 2.6 Statistics

Differences in relative accumulation of virus in RT-PCR positive plants and of hUTR/P1 hpRNA from transgene were determined by a Dunnet’s test after a one-way ANOVA.

## 3 Results

### 3.1 Plum pox virus RNA silencing-mediated resistance can be transmitted from transgenic rootstocks to wild-type scions

To evaluate the ability of transgenic rootstocks to confer PPV resistance in wild-type (wt) apricot scions, as a first approach (**Figure 1**), we grafted buds from PPV-infected wt apricots analyzing the PPV presence in the sprouting scions after an artificial winter (**Supplementary Figure 1**). Although many buds failed to sprout, probably due to the virus infection, PPV was not detected by RT-PCR in 75% and 40% of the scions sprouted from infected apricot buds grafted onto St5’-9 and St5’-1 rootstocks, respectively (**Table 1**) and in none of these rootstock lines. Conversely, PPV was detected in the new leaves of all susceptible St5’-7 rootstocks and all apricot scions. An intermediate PPV susceptibility behavior was observed for the tolerant St5’-6 line (**Table 1**). Collectively, more than 50% (five out of nine) of the wt scions sprouted from PPV-infected wt buds grafted onto the highly PPV-resistant transgenic rootstocks (St5’-9 and St5’-1) were PPV-free.

**Table 1:** RT-PCR evaluation of *plum pox virus* (PPV) in transgenic plum rootstocks grafted with PPV-infected apricot buds and on the sprouted apricot scions.

Transgenic rootstock lines	Number of plants^(a)^	Resistant status of transgenic rootstocks	Percentage of RT-PCR PPV-positive plants	
			Plum Rootstock	Apricot Scion
St5’-1	5	Resistant	0	60
ST5’-6	4	Intermediate resistance	50	100
St5’-7	5	Susceptible	100	100
St5’-9	4	Resistant	0	25

^(a)^A total of 118 buds were grafted, in similar number, onto the different plum rootstocks. However, most of them did not sprout or died shortly after sprouting. In the table are the surviving and evaluated grafted plants.

**Figure 2:**
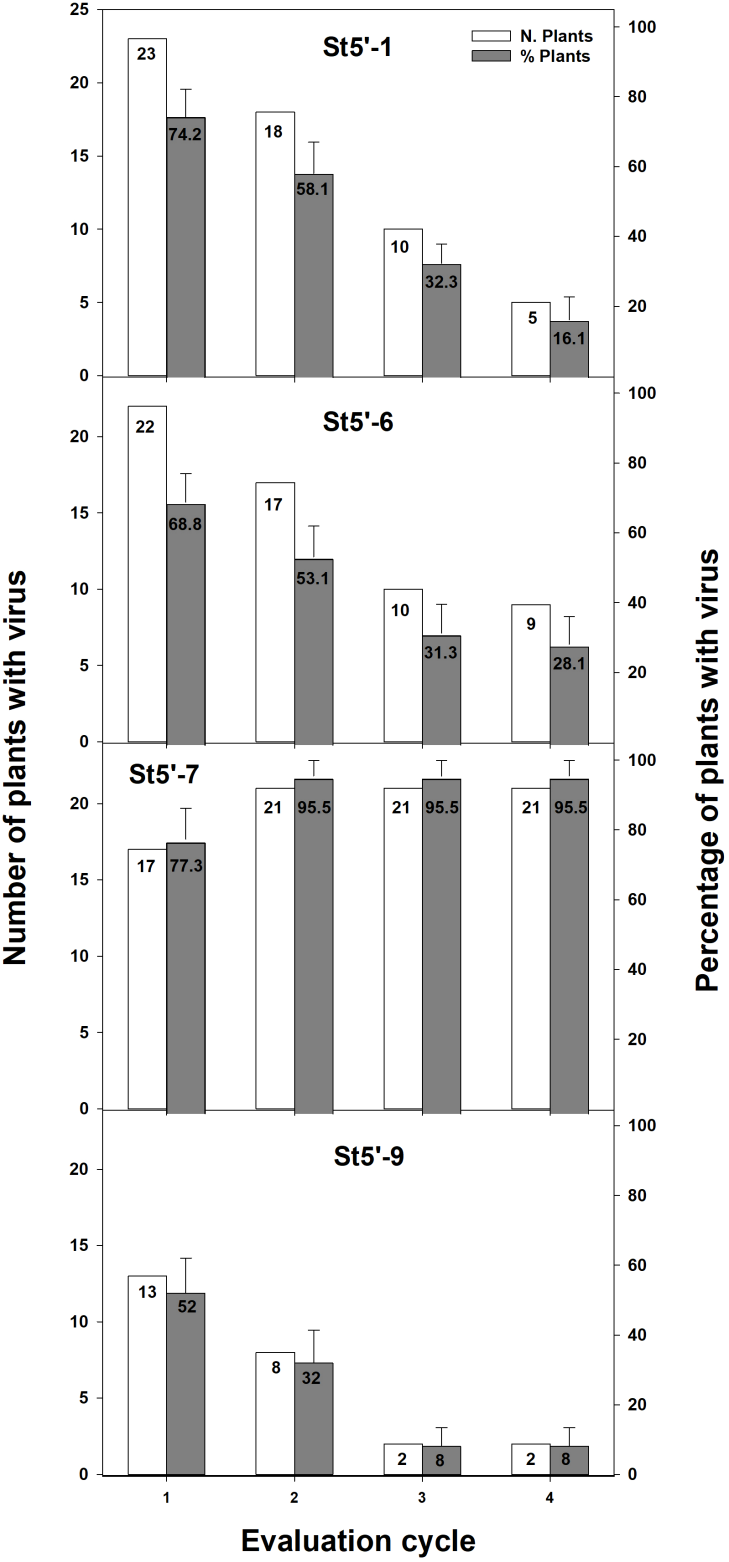
RT-PCR evaluation of PPV presence in the apricot scions grafted onto transgenic plum rootstocks during four consecutive dormancy-growth cycles. Total number of evaluated plants were 31 for St5’-1, 32 for St5’-6, 22 for St5’-7 and 25 for St5’-9.

To corroborate the above results, a different inoculum procedure was envisaged to ensure the analysis of a more significant number of plants (**Figure 1**). To this end, the four transgenic plum rootstocks were grafted whenever possible with two-three healthy buds. After an artificial winter in the cold chamber, the buds were forced to sprout by severely trimming the rootstocks plants, and scions were PPV-challenged by chip-budding (**Supplementary Figure 2**). PPV infection was evaluated by RT-PCR after an artificial winter followed by sprouting in the greenhouse (**Supplementary Figure 4**). When more than one bud sprouted on the same rootstock, all grafted apricots were evaluated independently. In those cases, the plant was considered resistant only when none of the scions was PPV-positive by RT-PCR. One hundred and ten wt apricots shoots grafted on transgenic rootstocks were analyzed (**Figure 2**). PPV was not detected in wt apricot scions of 48% of the St5’-9 rootstocks and 23% of the St5’-7 susceptible line (**Figure 2**). Twenty-eight plants were randomly chosen and re-evaluated at the beginning of the next cycle (about 2-3 weeks after the artificial winter) to confirm the data. We found that all results were identical except for one plant grafted onto a St5’-9 rootstock, one grafted onto a St5’-6, and two grafted onto a St5’-1 that switched from an RT-PCR positive reading to a negative one. The fact that in some RT-PCR positive scions, grafted onto PPV-resistant and -tolerant rootstocks, PPV was not detected more suggested a progressive recovery of wt scions from viral infection. To this end, all the PPV-positive plants in the first cycle were re-valuated after an additional cycle (**Figure 2**, second cycle). Notably, in five plants from each line St5’-1, St5’-6, and St5’-9, that were PPV-positive at the first cycle, the virus was not detected again. Conversely, no one of the PPV-positive apricots plants grafted on the susceptible St5’-7 line became virus free. In addition, four of the five PPV-negative plants of the susceptible St5’-7 line became PPV-positive in the second cycle bringing the infection efficiency to 95,5% (**Figure 2**). The artificially induced dormancy and greenhouse growth, followed by RT-PCR evaluation, was repeated for two additional cycles. In the fourth cycle, only 8%, 16%, and 28% of the wt apricots grafted on St5’-9, St5’-1, and St5’-6 were infected by PPV, respectively. The percentage of PPV resistance observed on the wt scions correlates well with the transgenic rootstocks’ degree of PPV (**Figure 2**) resistance (García-Almodóvar et al., 2015). Our data support the notion that the RNA-silencing-based PPV resistance (García-Almodóvar et al., 2015) can be transmitted from PPV-resistant plum rootstocks to wt apricot scions and that its efficiency is augmented after successive growth cycles.

### 3.2 Recover from virus infection after a second challenge with PPV occurs already within the same growing cycle

To further test the ability of the transgenic rootstocks to confer PPV-resistance in the wt scions, a group of apricot scions that were PPV-free in the previous cycle was challenged a second time with PPV and the presence of the virus evaluated twice within the same growing cycle (**Table 2**).

**Table 2:** RT-PCR evaluation of plum pox virus (PPV) at different times during the same growing cycle after a second challenge with the virus of apricot scions grafted onto sharka-resistant transgenic rootstocks.

Transgenic rootstock line	N° of plants challenged for a second time with PPV	RT-PCR + same cycle after 8 weeks	RT-PCR + same cycle after 13 weeks	Switch % from RT-PCR positive to negative
St5’-1	10	10	5	50
St5’-6	9	9	5	44.4
St5’-9	9	9	4	55.6

The first evaluation, using young leaves, was carried out eight weeks after chip-budding. RT-PCR analyses identified PPV in all plants. Notably, only five weeks later, thirteen weeks after re-infection, 44.4% to 55.6% of the wt plants, depending on the transgenic rootstock line, were virus-free (**Table 2**). The above data show that the PPV resistance conferred by the rootstocks was robust, already occurring within the same growing cycle and maintained in successive evaluation cycles.

### 3.3 PPV accumulates at a lower level in wild-type scions of the highly resistant transgenic rootstocks

**Figure 3:**
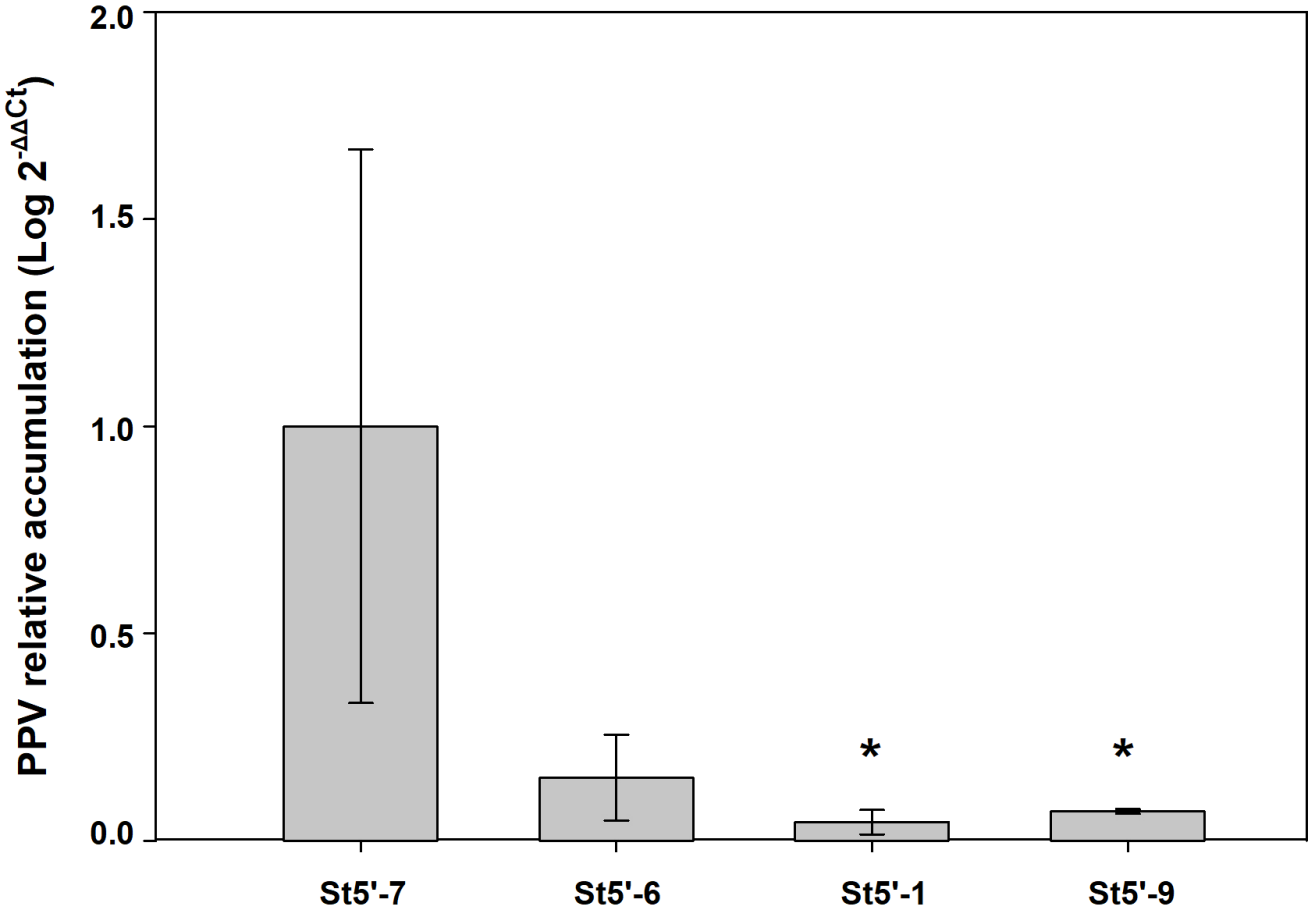
RT-qPCR quantification of plum pox virus (PPV) in virus-infected apricots grafted onto transgenic plum rootstocks that gave positive by RT-PCR to the virus presence in the last evaluation cycle. Each value is the mean from three biological replicates, each a pool from three plants. For each biological replicate six repetitions were run. Asterisks indicate significant differences (P<0.05) with the reference sample (apricots grafted onto the susceptible St5’-7 rootstock) according to a one-tail Dunnett’s test.

As shown in **Figure 2**, depending on the cycle taken into consideration and the transgenic rootstock line, a certain number of wt scions were infected by the virus. RT-qPCR analysis was conducted, during the last growing cycle evaluated, on a random subset of PPV-positive wt scions from the four transgenic rootstocks. The relative accumulation of PPV in the apricot scions grafted onto the resistant plum rootstocks St5’-1 and St5’-9 was significantly lower than those found in apricot scions grafted onto the susceptible St5’-7 line (**Figure 3**). In addition, apricots grafted onto the tolerant St5’-6 line had a lower relative accumulation of the virus than St5’-7, although differences were not significant according to a one-tail Dunnett’s test (**Figure 3**). The above data, together with those reported in **Figure 2** and **Table 2**, pointed out that the RNA silencing mechanism reduces the relative accumulation of virus progressively till eliminating the virus from the wt scions grafted on the transgenic resistant PPV plants.

### 3.4 Transgenic-derived 21-22 and/or 24-26 nt PPV siRNAs accumulate in wild-type apricot scions grafted on the PPV-resistant transgenic plum lines

The production of transgene-derived virus-specific siRNAs is a pre-requisite for RNA silencing to exert its virus-interference function. Moreover, the ability of the transgenic rootstocks to confer virus resistance to a grafted wt scion is dependent on the accumulation in the scions of the transgene-derived siRNAs above a certain threshold level (Sidorova et al., 2021). To this end, we evaluated and compared the presence of the h-UTRP1-derived PPV siRNAs in the four transgenic plum rootstocks, the PPV highly resistant St5’-1 and St5’-9, the tolerant St5’-6, and the susceptible St5’-7 and in wt healthy apricot scions grafted on them.

**Figure 4:**
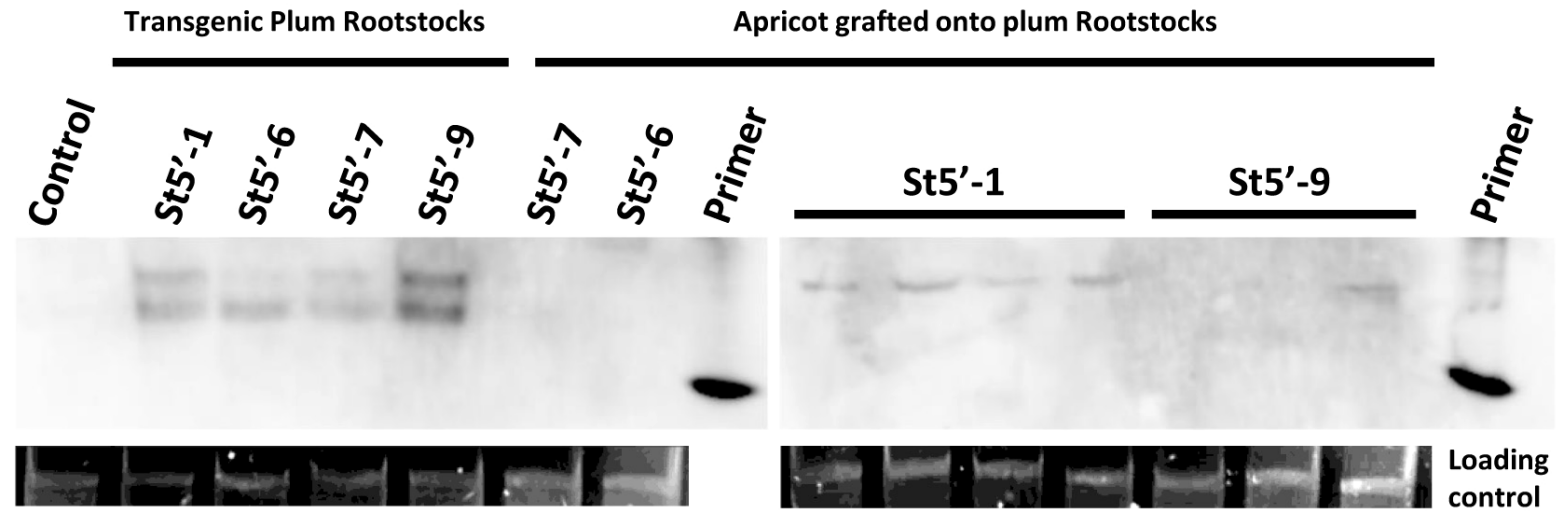
Northern blot detection of small interfering RNAs in transgenic plum rootstocks (samples taken from *in vitro* shoots) and grafted wild-type apricots after infection. The siRNAs were hybridized with a DIG-labeled RNA probe corresponding to the plum pox virus h-UTR/P1 cassette (Di Nicola et al., 2005). Primers are a 20 or 25-nucleotides-length DNA oligo (35Sdag5’-F and R). Control is RNA from wild type ‘Canino’ apricot. The loading control is total RNA.

Northern blot analysis identified two bands migrating slower than the 20 bp primer marker in the RNA extracted from all transgenic plum rootstock leaves. In particular, the highly resistant St5’-1 and St5’-9 plum lines and the tolerant St5’-6 line accumulated more PPV-specific 21-22 nt siRNAs compared to the PPV susceptible St5’-7 line (**Figure 4**). In addition, the St5’-6 rootstock accumulated 24-26 nt siRNAs. The detection limit of the northern blot did not permit to see the accumulation of siRNA, if any, in all the apricot scions. Leaves of apricot scions grafted onto most of the St5’-1 line (4 out of 7 of these plants), some of the St5’-9 and St5’-6 lines (2 out of 5 and 2 out of 6 plants, respectively) accumulated the 21-22 nt siRNAs alone or with 24-26 nt siRNAs, whereas 24-26 nt siRNAs were not detected in 7 plants analyzed from the apricot scions grafted onto the St5’-7 rootstock, but only the smaller 21-22 band was found in 3 of those plants (**Figure 4**). When seen, in the scions grafted onto the resistant St5’-1 and St5’-9 and the tolerant St5’-6 rootstocks, the signal for the 24-26 nt siRNAs was more intense than the 21-22 nt siRNAs indicating a preferential accumulation of the 24-26 nt siRNAs in the recipient scions that was not found in the scions grafted on the susceptible St5’-7 rootstock.

### 3.5 Quantification of hpRNA derived from the transgene expression is significantly lower in the resistant plum rootstocks than in the susceptible or tolerant plum lines

**Figure 5:**
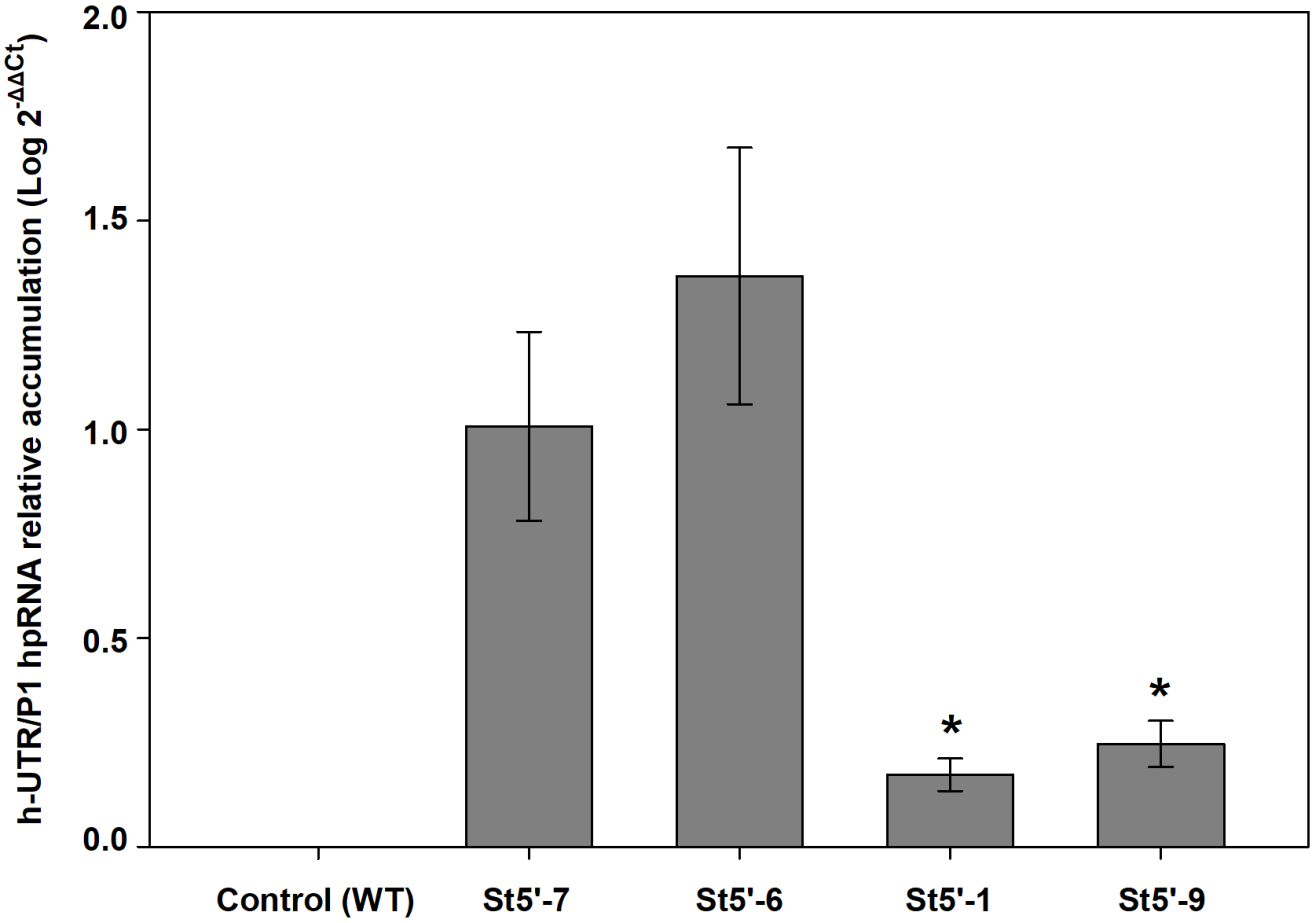
Relative quantification of transgene hpRNA in transgenic plum rootstocks. Asterisks indicate significant differences (P<0.05) with the reference sample (the susceptible St5’-7 rootstock) according to a one-tail Dunnet’s test.

Real time PCR quantification of the transgene hpRNA amount in leaves, collected from *in vitro* shoots of the different transgenic plum lines demonstrated a significantly lower amount in the resistant plum lines St5’-1 and St5’-9, used as rootstocks, than in the susceptible plum line St5’-7 and the tolerant St5’-6 (**Figure 5**). No significant differences were found between St5’-7 and St5’-6 lines in hpRNA quantification

## 4 Discussion

The success of transgene-derived siRNA transmission through grafting would be of practical importance in horticulture. Grafting wild-type scions onto transgenic silenced rootstocks could improve individual traits of well-established non-transgenic cultivars, particularly for those recalcitrant to regeneration or transformation. It would be instrumental in the case of *Prunus* species that are very difficult to transform, such as apricot (Petri et al., 2015) or peach (Ricci et al., 2020). Notably, it is expected that public concerns about using transgenic plants should be mitigated by the lack of transgenes spreading by outcrossing coupled with the consumption of non-transgenic edible parts of the plant.

Although encouraging, our first data (**Table 1**) studying the transmission of PPV resistance from transgenic plum rootstocks to wt apricot scions were limited in broadness due to the reduced sprouting of the wt apricot PPV-infected buds, possibly due to a high inoculum pressure. A similar technical limitation was also reported by Zhao and Song (2014), which showed that grafting PNRSV infected buds onto sweet cherries results in the death of the buds.

To overcome the above limitation, we adopted the chip budding technique. This inoculum procedure permitted us to study the resistance behavior of one hundred and ten grafted wt apricots challenged with a PPV-D isolate.

The data clearly shows an increase with time in the number of PPV-free plants as evaluated by the RT-PCR analysis (**Figure 2**). In addition, the low percentage of PPV-infected apricot plants grafted onto resistant rootstocks accumulated significantly fewer amounts of the virus when compared to the susceptible rootstock (**Figure 3**). Notably, when non-transgenic scions that recovered from viral infection were re-challenged with PPV, the RNA silencing effectively eliminated the virus within the same growth cycle in about 50% of scions (**Table 2**). The ability to recover from virus infection is a peculiar characteristic of the RNA silencing-mediated resistance occurring both during natural viral infections (Covey et al., 1997; Ratcliff et al., 1997) and in herbaceous and woody transgenic plants (Ravelonandro et al., 1993; Dougherty et al., 1994; Lindbo and Dougherty, 2005; García-Almodóvar et al., 2015).

To the best of our knowledge, there are only four previous papers intending induced silencing from a rootstock to a scion in woody plants, with contradictory results. In apples, transgenic rootstock-mediated silencing in the scions was shown to occur for a *gusA* transgene but not for an endogenous anthocyanidin synthase gene. Additionally, nor the transgene nor the endogen was silenced when the grafting experiment were conducted in the greenhouse (Flachowsky et al., 2012). Authors hypothesized that lignification might influence cell-to-cell transport of siRNAs in living cells, thus explaining the lack of silencing effect.

In a recent paper (Sidorova et al., 2021), two PPV-resistant transgenic plum cultivars transformed with a hairpin to silence the virus capsid gene were evaluated for their capacity to transfer the PPV resistance character to the wild-type grafts. They found that transgenic rootstocks remained virus free but could not protect the scion due to the lack of an efficient transfer of transgene-derived siRNAs from the rootstocks to the scions. However, scions accumulated specific endogen sRNAs characteristic of the rootstocks (Sidorova et al., 2021). Similar results were found using the PPV-resistant transgenic plums ‘Honeysweet’ or B-14, transformed with the PPV-CP gene (Ravelonandro and Briard, 2023). Conversely, Zhao and Song (2014) showed that PNRSV-hpRNA-derived siRNAs were transmitted up to 1.2 m from the transgenic sweet cherry rootstocks to the non-transgenic scions conferring enhanced PNRSV resistance.

Variable results were also obtained in works dealing with grafting-mediated virus resistance in horticultural species. Bai et al. (2016) showed that 66.7% to 83.3% of non-transgenic tomatoes were highly resistant to CMV. In tobacco, detached leaves from scions grafted on transgenic tobacco silenced for the endogenous *NtTOM1* and *NtTOM3* genes were shown to accumulate fewer tobamoviruses than the control plants (Ali et al., 2013). Similarly, *Nicotiana benthamiana* transgenic plants expressing a hairpin designed to silence PSTVd produced only attenuation of viral infection (Kasai et al., 2013). The contradictory data can be attributed to differences between plant species, the transgenic construct used and/or the targeted sequence (exogenous infecting virus or endogenous gene transcripts).

Our data do not support the hypothesis of lignification as the primary cause of the lack of RNA silencing spreading from rootstocks to scions (Flachowsky et al., 2012; Sidorova et al., 2021). Apricot scions and plum rootstocks were well-lignified during the four years that the experiment lasted (from rooting and acclimatizing the rootstocks, grafting apricots, and infecting them by chip-budding to the final evaluation).

The siRNAs analyses identified the accumulation of the PPV UTR/P1 24-26 nt siRNAs in apricot scions grafted on the PPV resistant but not onto the susceptible rootstock (**Figure 4**). In contrast, faintly amount of 21-22 nt siRNAs were detected in the apricot scions on resistant rootstocks but more clearly seen on St5’-7 scions (**Figure 4**), suggesting that the 24-26 nt siRNAs can be: a) preferably transported over a long distance (Hamilton et al., 2002; Molnar et al., 2010); b) less prone to degradation or; c) less consumed by AGO in the traversed and recipient cells (Voinnet, 2022). The first PPV-resistant transgenic *Prunus* was the plum C5 (‘Honeysweet’) that strongly silence the coat protein virus gene by PTGS (Scorza et al., 2001). When studying the molecular mechanisms associated with the resistance to sharka of C5 plum, 24 nt siRNAs was related to systemic silencing (Kundu et al., 2008). In particular, they were present only in resistant C5 plants but not in susceptible ones nor in C5 plants showing middle sharka symptoms. The evidence that the tolerant and resistant plum rootstocks could protect the apricot scions, and that 24-26 nt siRNAs were only found in these scions but never in those grafted onto susceptible St5’-7 line, agrees with results found in ‘Honeysweet’ plum.

Different works suggest that all siRNA classes (21, 22, and 24 nt long siRNAs) are mobile (Devers et al., 2020), with the 22 nt siRNAs having a pivotal role in the siRNAs signal amplification and translational repression (Chen et al., 2010; Cuperus et al., 2010; Wu et al., 2020). Trans-grafting movement of siRNAs is not a simple concentration dependent diffusion process, but probably requires a selective sRNA sorting mechanism and recent studies suggest that it might be dictated by sRNA biosynthetic pathways, sRNA sizes, sequence features such as 5’ nucleotide, or selective RNA-binding protein partners (Kong et al., 2022). It will be interesting to evaluate the amounts and nature/diversity of 5′-nucleotide identities/sizes of siRNAs accumulating in the grafted apricot scions and transgenic rootstocks using a more sensible and specific technique.

Northern blot analysis identified, in addition to the siRNA in PPV-resistant plum, transgene-derived UTR/P1 siRNAs in all transgenic rootstocks independently on the level of PPV resistance, indicating that their accumulation is necessary, but not sufficient, to assure efficient PPV interference. These data agree with those obtained by López et al. (2010) in Mexican lime transformed with sense, antisense, and intron-hairpin cDNAs from viral sequences and with data from tobacco (Alburquerque et al., 2012) or plum (Alburquerque et al., 2017) transformed with a chimerical ihp-transgene designed to silence *Agrobacterium* oncogenes *iaaM* and *ipt*. In those works, all resistant lines accumulated transgene-derived siRNAs, but this was not necessarily associated with resistance to citrus tristeza virus (López et al., 2010) or crown gall (Alburquerque et al., 2012; Alburquerque et al., 2017). Therefore, a lower amount of hpRNA seems to be better correlated with resistance. Although this could be due to lower expression or higher degradation, it seems logical to think that resistance is related to a more efficient degradation of the hpRNA (dsRNA) being recruited by DICER for subsequent PTGS of the target sequence.

Previous studies showed that transgenic C5 plants were resistant to PPV when exposed to natural viruliferous aphids while accumulating low-level PPV near the graft junction if graft-inoculated (Malinowski et al., 2006). Based on the C5 plants data, we expected that the apricots grafted onto the PPV-resistant plum lines should also be resistant to PPV infection under natural field conditions. Importantly, since the PPV-derived h-UTR/P1 construct present in transgenic plum rootstocks was derived from a PPV isolate belonging to the M strain while the plants were challenged with a PPV-D isolate, it suggests that the resistance observed should be extended to, at the very least, the viral isolates of the two most important and widespread PPV strains.

This work aimed to study the possible transfer of sharka resistance from a transgenic plum rootstock to apricot scions. For this purpose, grafted plants were infected by chip budding and evaluated by RT-PCR during four growing seasons. A schematic representation of the process, lasting a total of almost 4 years is shown in **Figure 1**. As conclusion, the results demonstrate for the first time that PPV-resistant transgenic plums can effectively confer sharka resistance in grafted non-transgenic apricots scions. It is expected that using transgenic rootstocks can mitigate public concerns about transgene dispersions and eating transgenic food. Additionally, it would allow conferring resistance to sharka to recalcitrant- to-transformation cultivars or even important species such as peach (Ricci et al., 2020).

Additional studies on the long-distance movement of the RNA silencing signal are required to understand how broadly applicable this technique is to modulate the phenotype of wild-type grafted scions in woody plants. Uncovering the mechanism of sRNA selection for trans-grafting transport will potentially enhance success in designing artificial sRNAs to control plant disease.

## Data availability statement

The original contributions presented in the study are included in the article/**Supplementary Material**. Further inquiries can be directed to the corresponding author.

## Author contributions

NA and CP-C did most of the experimental work, help analyzing and preparing data and revised the manuscript. LF technically assisted maintaining plants and doing some of the qPCR analysis. VI provided the constructions and revised the manuscript and LB designed the work, help in the experimental work, help with data analyses and wrote the manuscript. All authors contributed to the article and approved the submitted version.
