# Supplementary material for: Trans-grafting plum pox virus resistance from transgenic plum rootstocks to apricot scions
Source: Front Plant Sci. 2023 Sep 27;14:1216217. doi: 10.3389/fpls.2023.1216217 (PMC10565502; doi:10.3389/fpls.2023.1216217)
Supplement: Supplementary file 1 [file DataSheet_1.docx]

Supplementary Material

Effective transfer of plum pox virus resistance from transgenic plum rootstocks to apricot scions

**Alburquerque, N.*^1^; Pérez-Caselles, C. *^1^; Faize, L. ^1^; Ilardi, V. ^2^ and Burgos, L. ^1^**

*** Correspondence:** Dr. L. Burgos: burgos@cebas.csic.es

**
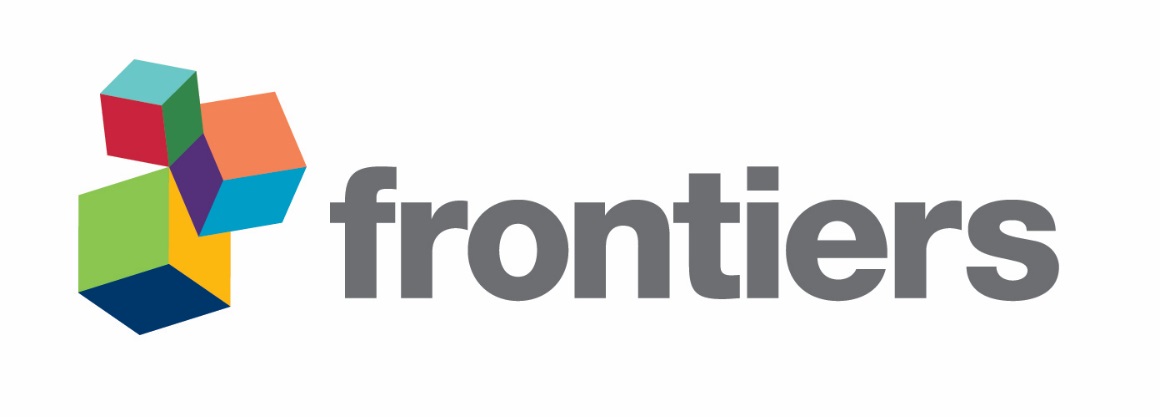
**

**Supplementary Figure 1.** Apricot grafted onto a transgenic plum rootstock. (A) Apricot bud grafted sprouting after artificial winter. (B) Heavily infected apricot scion grafted onto the transgenic susceptible plum line St5’-7 and (C) healthy apricot grafted onto the resistant plum rootstocks. (D) Detail of an apricot grafted plant (ag) after trimming the plum rootstock (pr) where can be seen the point of chip-budding infection (cb).


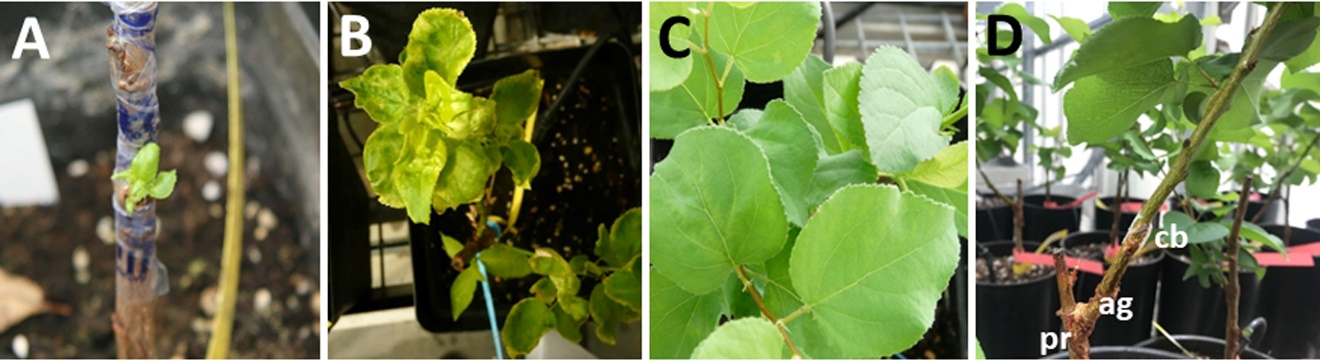


**Supplementary Figure 2.** Chip budding technique used to infect apricot scions with sharka. A cut in the apricot scion (1) allows the introduction of a piece of bark from an infected GF305 new growth (2) within the apricot scion (3). Grafting is wrapped with parafilm to avoid desiccation (4) and it is maintained until it dries out and falls (5).


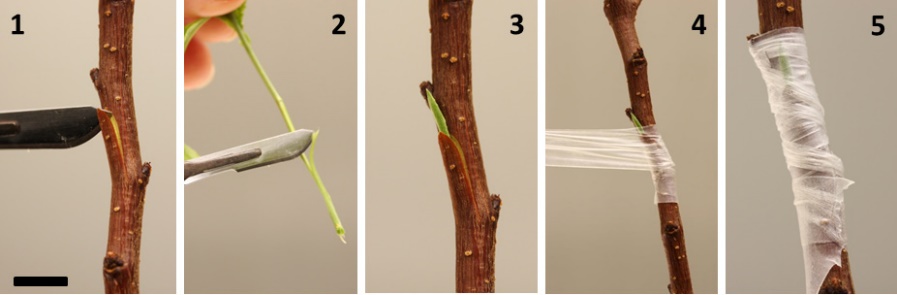


**Supplementary Figure 3.** Schematic representation of the *h-UTR/P1* T-DNA showing the position of primers used in this study.


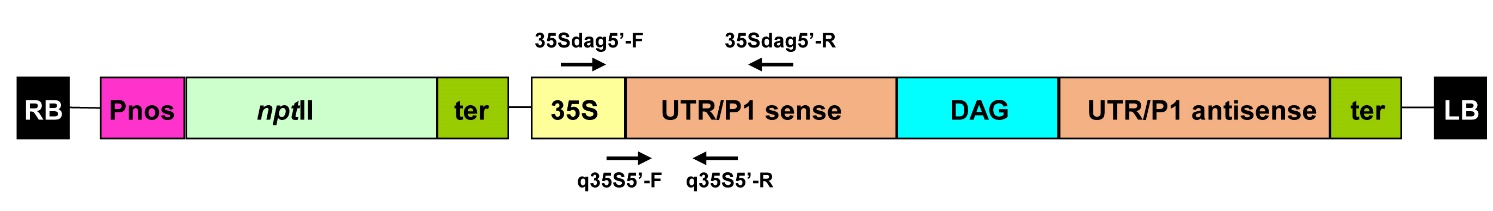


**Supplementary Figure 4.** Electrophoretic analysis of PCR products for PPV detection. L: DNA ladder 100 pb (New England Biolabs, Cat. no. N3231L). Lanes 1-10: apricot scion grafted onto different St5’ transgenic rootstocks. Lane 1 and 2: onto St5’-7 plants. Lanes 3 and 4: onto St5’-6 plants. Lanes 5-7 onto St5’-1 plants. Lanes 5-7 onto St5’-9 plants. Lane 10 H2O.


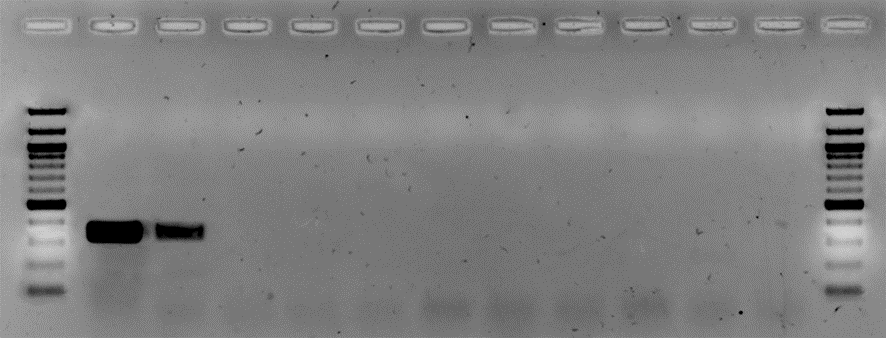


**L**

**1**

**2**

**4**

**3**

**5**

**6**

**7**

**8**

**9**

**313 bp**

**10**

**L**

**11**
